# Evaluation of Telemedicine awareness, knowledge, attitude, skill levels of physicians and students

**DOI:** 10.1093/eurpub/ckac130.064

**Published:** 2022-10-25

**Authors:** A Mutlu, A Kilinc, L Ozcan, M Tepetas, S Metintas, MF Onsuz

**Affiliations:** Department of Public Health, Osmangazi University Faculty of Medicine, Eskisehir, Turkey

## Abstract

**Background:**

The increase in the use of information and communication technologies around the world brings about developments and changes in the provision of health services. It is accepted that telemedicine applications will facilitate health services for patients and health personnel. This study aimed to evaluate the telemedicine awareness, knowledge, attitude and skill levels of physicians and medical school students.

**Methods:**

This cross-sectional study was conducted between July and August 2021. A questionnaire form was prepared using the relevant literature then filled online by the participants. Telemedicine Awareness, Knowledge, Attitudes and Skills Questionnaire was used in the research. The universe of the research consisted of medical faculty students and physicians across Turkey. In the study, 933 people were reached by using the purposeful snowball sampling method, one of the non-probability sampling methods. Mann Whitney-U test, Kruskal Wallis test, Spearman correlation analysis and Multiple Linear Regression analysis were used.

**Results:**

Of the participants, 442 (47.4%) were female, 497 (53.3%) were medical students, and 436 (46.7%) were physicians. Their ages ranged from 18 to 59, with a mean of 28.0±8.8 years. The median (min-max) scores obtained from the Telemedicine Awareness (TA) sub-section were 18(0-24), 51.3% of them had scores above the median score. TA sub-section had a moderate positive correlation (r = 0.559, p < 0.001) with knowledge sub-section and a weak positive correlation (r = 0.208, p < 0.001) with skill level sub-section. TA scores were higher among men, physicians and those who want to participate in a telemedicine training program. TA increased as the level of computer and internet knowledge increased (F = 29.171, R2=0.197, p < 0.001).

**Conclusions:**

It can be said that TA is at a moderate level among medical students and physicians in Turkey. TA increased as the level of computer and internet knowledge, telemedicine knowledge and skills increased.

**Key messages:**

- It may be beneficial to add telemedicine education to the medical school curriculum.
- For physicians in working life, it is recommended to include telemedicine applications in the scope of continuing medical education.

